# Genetic and functional analysis of two missense *DUOX2* mutations in congenital hypothyroidism and goiter

**DOI:** 10.18632/oncotarget.10525

**Published:** 2016-07-11

**Authors:** Shiguo Liu, Wenhui Zhang, Liqin Zhang, Hui Zou, Kunna Lu, Qiang Li, Hongfei Xia, Shengli Yan, Xu Ma

**Affiliations:** ^1^ Prenatal Diagnosis Center, The Affiliated Hospital of Qingdao University, Qingdao, 266003, China; ^2^ Genetic Laboratory, The Affiliated Hospital of Qingdao University, Qingdao, 266003, China; ^3^ Endocrinology Department, The Affiliated Hospital of Qingdao University, Qingdao, 266003, China; ^4^ Endocrinology Department, Liaocheng People's Hospital, Liaocheng, 252002, China; ^5^ Neonatal Screening Center, Qingdao Women & Children Medical Healthcare Center, Qingdao, 266003, China; ^6^ Neonatal Screening Center, Jinan Women & Children Medical Healthcare Center, Jinan, 250000, China; ^7^ Endocrinology Department, The Affiliated Hospital of Taishan Medical College, Taian, 271000, China; ^8^ Andrology Department, The Affiliated Hospital of Qingdao University, Qingdao, 266003, China; ^9^ Graduate School, Peking Union Medical College, Beijing, 100000, China; ^10^ National Research Institute for Family Planning, Beijing, 100081, China; ^11^ World Health Organization Collaborating Centre for Research in Human Reproduction, Beijing, 100000, China

**Keywords:** congenital hypothyroidism, DUOX2, DUOXA2, mutation, function

## Abstract

Mutations in the dual oxidase 2 gene (*DUOX2*) impair hydrogen peroxide (H_2_O_2_) production and cause dyshormonogenesis. In addition, these mutations have been implicated in autosomal recessive congenital hypothyroidism (CH) with goiter. In this study, we identified *DUOX2* mutations that were causative for CH and explored the effects of these mutations on DUOX2 function. Blood samples were collected from 10 infants born with CH and goiter to unrelated parents. We extracted genomic DNA and sequenced all exons by polymerase chain reaction direct sequencing. The effects of *DUOX2* mutations were characterized by H_2_O_2_ production assays and cycloheximide (CHX) chase experiments. Sequence analysis revealed one novel *DUOX2* mutation and one known *DUOX2* mutation in unrelated families: c.1060C>T (p.R354W) and c.3616 G>A (p.A1206T). Both mutations impaired H_2_O_2_ production. CHX chase experiments demonstrated the *DUOX2* mutants had shorter half-lives and degraded more rapidly than wild-type *DUOX2.* Our study identified two novel *DUOX2* mutations in Chinese patients with CH and goiter, which were responsible for the deficit in the organification process.

## INTRODUCTION

Congenital hypothyroidism (CH) is one of the most common neonatal endocrine disorders, presenting with abnormal growth and intellectual impairment. Symptoms are caused by reduced thyroid function. CH occurs in 1 in 3000–4000 births and affects twice as many females as males [1, 2]. Without prompt thyroid hormone replacement, the physical and mental disability can become permanent. Clinical manifestations of CH mainly include poor feeding, prolonged jaundice, edematous, umbilical hernia, and dry skin. The majority of cases are sporadic, while hereditary cases show classical Mendelian autosomal recessive inheritance [3]. CH patients can be divided into two groups: about 85% of cases are caused by thyroid dysgenesis, including agenesis (22–42%), ectopy (35–42%), and hypoplasia (24–36%) [4], and the remaining 15% are associated with dyshormonogenesis. To date, a variety of gene mutations have been associated with thyroid hormone synthesis, such as thyroglobulin (*TG*) [5], thyroperoxidase (*TPO*) [6], sodium/iodide symporter (*NIS*) [7], dual oxidase 2 (*DUOX2*) [8], DUOX maturation factor 2 (*DUOXA2*) [9], pendrin (PDS) [10], and iodotyrosine deiodinase (*DEHAL1*) [11].

A central part of thyroid hormone synthesis is iodide organification, which requires thyroid peroxidase (*TPO*) and hydrogen peroxide (H_2_O_2_). H_2_O_2_ is used as a substrate in the iodination of tyrosine residues and the coupling of iodotyrosine residues to TG [12]. The DUOX1, DUOX2, DUOXA1 and DUOXA2 proteins have recently been identified as components of the H_2_O_2_ generation system in the thyroid. Among them, DUOX1 and DUOX2 are essential enzymes in the production of H_2_O_2_. DUOXA1 and DUOXA2 enable the DUOX proteins to translocate to the plasma membrane and become fully active [13]. *DUOX1* and *DUOX2* encode two very similar proteins that are inserted into the apical membrane of the thyroid follicular cell. *DUOX2* expression is at least five times higher than *DUOX1* [14, 15]. Defective H_2_O_2_ production causes CH. Defects in DUOX2 cause severe, permanent CH due to complete disruption of thyroid hormone synthesis or milder, transient hypothyroidism caused by insufficient quantities of thyroid hormones [13].

*DUOX2* is located on chromosome 15q15.3 and consists of 34 exons, which of its 33 exons encode a 6376 nucleotide mRNA [16, 17]. The DUOX2 protein, a 1548-amino-acid polypeptide including a 26-amino-acid signal peptide, localizes to the apical membrane of thyrocytes and is involved in the Ca^2+^/reduced nicotinamide adenine dinucleotide phosphate (NADPH)-dependent generation of H_2_O_2_ [18]. The structure of these proteins includes seven putative transmembrane domains, four NADPH-binding sites, one flavin adenine dinucleotide (FAD)-binding site, and two EF-hand motifs that putatively control enzymatic activity through calcium binding [19].

The aim of this study was to screen *DUOX2* mutations in 10 unrelated Chinese children with CH and goiter by standard polymerase chain reaction (PCR)-based sequencing and clarify genotype–phenotype relationships. We also examined the functional effect of the identified mutations on DUOX2 at the molecular level.

## RESULTS

### Screening of mutations

**Figure 1:**
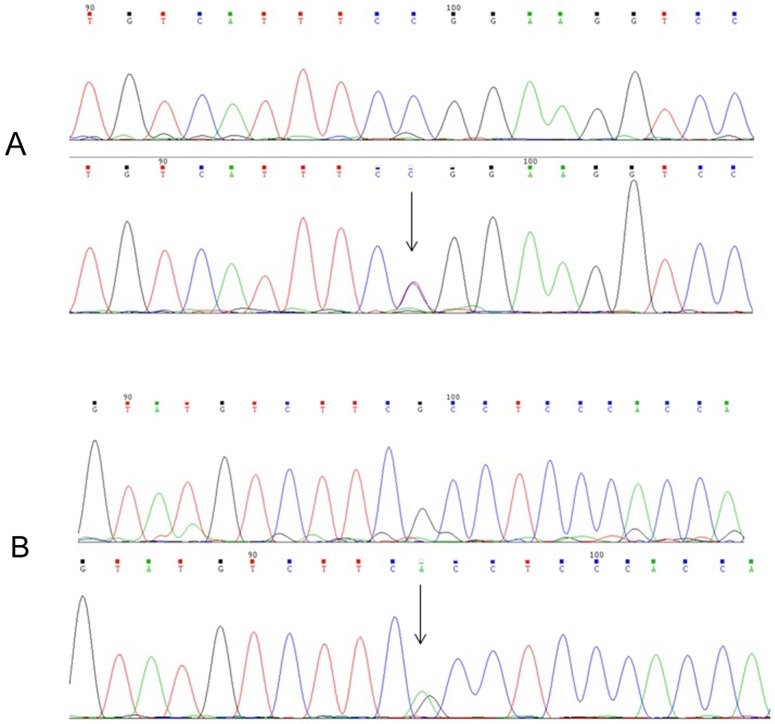
Partial sequence of exon 10 and exon 28 in the DUOX2 from WT(top) and mutational individuals (below) **A.** Arrowhead indicates the heterozygous C and T at nucleotide 1060 in an affected patient; **B.** Arrowhead indicates the heterozygous G and A at nucleotide 3616 in an affected patient.

Direct sequencing of *DUOX2* from 10 unrelated patients revealed two heterozygous missense mutations in two patients. One was a novel missense mutation (c.1060C>T), which results in an arginine to tryptophan substitution at codon 354 in exon 10 (p.R354W, Figure 1A). The other was a known missense variation (c.3616G>A), which predicts an alanine to threonine substitution at codon 1206 in exon 28 (p.A1206T, Figure 1B). However, no mutation was found in the remaining eight patients. Deleterious effects were predicted by Polymorphism Phenotyping v2 software (Polyphen) and Sorting intolerant from tolerant (SIFT) software. Polyphen predicts the possible impact of an amino acid substitution on the structure and function of a human protein using physical considerations and SIFT predicts whether an amino acid substitution affects protein function. Damaging effects on the DUOX2 protein were confirmed for both *DUOX2* mutation as follows: p.R354W: SIFT score 0.01, PolyPhen-2 score 0.98; p.A1206T: SIFT score 0, PolyPhen-2 score 1. We did not find these two variants in 100 Chinese healthy controls. Mutation segregation with the phenotype within the family was not performed because data was lacking.

### Clinical data

Patient 1 with a p.R354W mutation was a male infant. He was born after full-term gestation by cesarean delivery from unrelated parents. His birth weight was 2,800 g. There was no family history of thyroid disease. Neonatal screening showed a high level of TSH (187.6 μIU/ml; upper limit, 20 μIU/ml), so he was recalled at the age of 23 days for further evaluation. At that time, his body weight was 3,600 g and his height/length was 53 cm. Biochemical evaluation showed a serum TSH level of 218 uIU/ml and a FT_4_ level of 4.2 pmol/L. A 99mTc thyroid scan confirmed goiter (Left lobe:12×10mm, Right lobe 13×10mm) and the perchlorate discharge test was positive (iodine uptake rate >30.51%). Levothyroxine (L-T_4_) replacement therapy was started immediately at an initial dose of 8.3 μg/kg per day. Replacement therapy was modified during follow-up according to clinical and hormonal evaluations to maintain normal serum TSH and FT_4_ levels. At 2.5 years of age, TSH, FT_4_, and FT_3_ concentrations were normal following a temporary withdrawal of L-T_4_ therapy for 4 weeks. Therefore, he was diagnosed with transient CH. The patient is now 4.5 years old. His physical and mental development is normal.

Patient 2 (P2), was a female subject with a p.A1206T mutation, born by cesarean delivery after full-term gestation to unrelated parents. Her birth weight was 2,650 g. There were no abnormal physical findings until neonatal screening at the age of 6 days, which revealed a high level of TSH (141.5 μIU/ml). The patient was diagnosed with CH according to the neonatal screening program. At the age of 21 days, the patient was recalled to confirm the test results, which showed a high TSH level (186.3 μIU/ml) and a low FT4 level (5.8 pmol/L). At the same time, a 99mTc thyroid scan showed a normally shaped orthotopic but mildly enlarged thyroid gland. L-T_4_ replacement therapy was started immediately at an initial dose of 8.8 ug/kg per day. The dose was adjusted according to clinical and hormonal evaluations to maintain normal serum TSH, FT_4_, and FT_3_ levels. When she was 2 years old, concentrations of TSH, FT_4_, and FT_3_ were normal following a temporary withdrawal of L-T_4_ therapy for 4 weeks. The patient is now 6 years old and her physical and mental development is normal, with no evidence of goiter.

### H_2_O_2_ production assay

**Figure 2:**
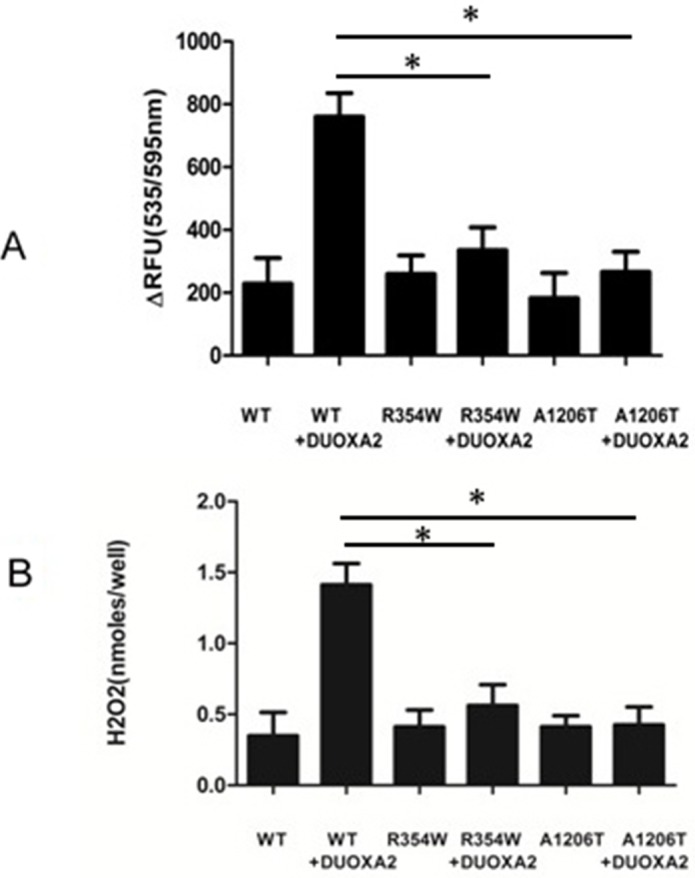
Showed H2O2 generation of cells transfected with the indicated expression vectors The total amount of plasmids pertransfection was kept constant by adjusting with empty vector. **A.** RFU shown the changes in resorufin fluorescence vs. the baseline of empty vector transfected cells. **B.** Nanomoles of H2O2 was converted from fluorescence intensity using a calibration curve. (n=3, ^*^p<0.05).

H_2_O_2_ generation was measured after either *DUOX2* alone or both *DUOX2* and *DUOXA2* were expressed in 293T cells (Figure 2). Transfection of *DUOX2* alone did not increase H_2_O_2_ production compared with nontransfected cells. The co-transfection of WT *DUOX2* with *DUOXA2* permits to DUOX2 to be active as indicated by the significant amounts of H2O2 released from the cells. Conversely, co-transfection of p.R354W and p.A1206T *DUOX2* even in the presence of *DUOXA2* did not show any activity, suggesting that these mutants were able to impair the functional activity of the H_2_O_2_-generating system.

### CHX chase experiments

**Figure 3:**
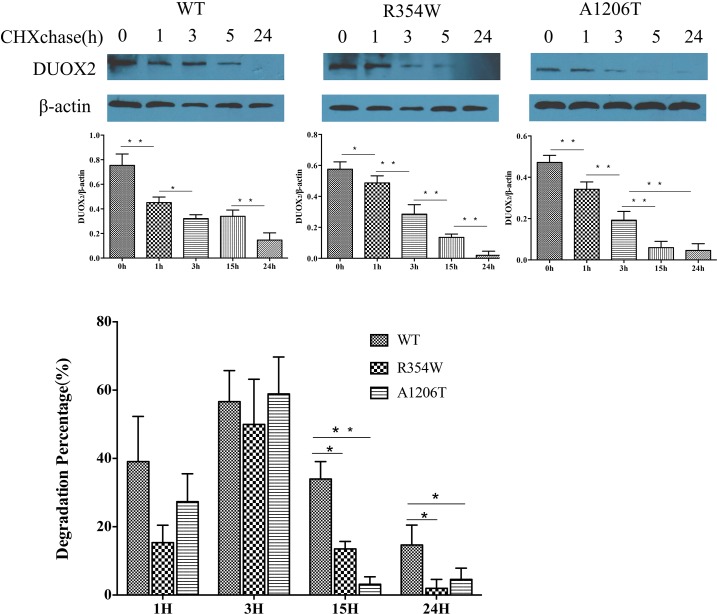
Cycloheximide(CHX) chase experiments 100 μg/mL CHX was added to the medium of Hela transfected with DUOXA2 and either WT or one of the mutations DUOX2 expression plasmids. Western blot analysis showed the expression of DUOX2 (175kda) and β-actin(43kda). The expression was quantified densitometrically as the ratio of DUOX2/β-actin.(n=3, ^*^p<0.05,^**^p<0.01).

Twenty-four hours after transfection with the indicated expression vectors, protein expression was inhibited by addition of CHX to the medium. Cell lysates were prepared at the indicated times thereafter, and equivalent amounts of total protein were immunoblotted. Compared with the WT DUOX2 proteins at the beginning of the incubation period (0 hours), *DUOX2* mutants had shorter half-lives and degraded more rapidly (Figure 3A). One hour and 3 hours after transfection with the indicated expression vectors, there was no significant difference in the degradation of DUOX2 proteins. However, 15 hours and 24 hours after transfection, the degradation of both DUOX2 mutants was higher compared with the WT group (Figure 3B).

## DISCUSSION

The generation of hydrogen peroxide is a critical step in the synthesis of thyroid hormones. Mutation of *DUOX2* causes hypothyroidism because of insufficient H_2_O_2_ production. DUOX proteins require DUOX maturation factors (DUOXA1 or DUOXA2) for the proper translocation of DUOX from the endoplasmic reticulum to the apical plasma membrane, where H_2_O_2_ production takes place. Since Moreno et al. reported the first *DUOX2* mutation in 2002 [20], at least 35 more *DUOX2* mutations have been identified, including missense mutations, nonsense mutations, frame shifts, and splice-site mutations [21, 22].

Grasberger used a *DUOXA2* reconstituted system to characterize natural *DUOX2* missense variants (p.Q36H, p.R376W, p.D506N) at the molecular level and analyze their impacts on H_2_O_2_ generation, trafficking, stability, folding and DUOXA2 interactions [23]. The p.Q36H and p. R376W mutations completely prevented translocation of DUOX2 to the cell surface. The p.D506N mutant displayed a partial deficiency phenotype with reduced surface expression but normal intrinsic H_2_O_2_-producing activity. The p.R376W [24] and p.Q36H [25] mutations were reported in permanent CH patients, while p.D506N [26] was found in temporary CH patients. Different *DUOX2* mutations cause completely different CH phenotypes. *In vitro* experiments have revealed obvious pathogenic functions for some and none for others.

In our study, we described two missense mutations—p.R354W and p.A1206T in *DUOX2*. The novel mutation p.R354W is located in the extracellular peroxidase-like domain, and the known p.A1206T mutation is located in the 5th transmembrane helix segments. p.R354 and p.A1206 are highly conserved in other mammalian species and p.R354W and p.A1206T were not identified in 200 alleles from healthy subjects. The H_2_O_2_ production assay showed no impairment in H_2_O_2_-generating activity by WT DUOX2 protein or mutant proteins. Coexpression of WT proteins with DUOXA2 in HeLa cells significantly reconstituted H_2_O_2_ production. Mutant proteins were still unable to produce H_2_O_2_, which corresponded with the iodine organification defect observed in patients. We also found that mutant DUOX2 degraded faster than mature WT DUOX2.

Each patient had an increased TSH and decreased T4 serum level after birth. The only clinical sign of hypothyroidism was goiter. Euthyrox treatment was started immediately after the diagnosis of CH, and was continued until 2 years of age. When serum FT3 and FT4 levels had returned to normal, the growth and development were normal. This demonstrated that the phenotypes of the two affected patients were both transient and mild and transient hypothyroidism was diagnosed. We previously identified novel mutation p.A1206T in two unrelated patients with transient hypothyroidism [27]. This is compatible with findings of Moreno, who reported that biallelic mutations cause permanent hypothyroidism, whereas monoallelic mutations cause transient hypothyroidism [20]. However, studies have reported biallelic inactivating *DUOX2* mutations in cases of mild hypothyroidism. Tonacchera identified a novel compound heterozygous mutation p.S911L/p.C1052Y in a child with CH and a eutopic thyroid gland [28]. Hoste [29] also reported a novel genetic defect (c.4552G>A, p.Gly1518Ser) together with deletion of exons 26–33 in a French-Canadian patient with transient CH and functional studies showed complete inactivation of DUOX2. Moreover, monoallelic permanent cases have indicated the heterogeneity of *DUOX2* mutations and suggested the possible involvement of multiple gene defects or epigenetic mechanisms [30, 31]. De Marco et al. revealed a *DUOX2* deletion mutation (p.S965fsX994) in three children with subclinical hypothyroidism and known functional impairment of H_2_O_2_ production, confirming that monoallelic mutations of DUOX2 protein can cause different phenotypes [30]. Muzza et al. found a p.S965fsX994 mutation in *DUOX2* in a patient with permanent CH and PIOD [31]. These studies provide further evidence that the transient or persistent nature of the hypothyroid phenotype is not directly related to the number of inactivated *DUOX2* alleles.

With the increasing number of reported *DUOX2* mutations, genotype–phenotype correlations have become more complex than initially anticipated. The clinical spectrum of patients with *DUOX2* mutation ranges from moderate to severe goitrous hypothyroidism. Yoshizawa-Ogasawara described two unrelated Japanese girls with a p.G488R mutation [32]. One patient was a compound heterozygote for p.L479SfsX3/p.G488R and the other was homozygous for p.G488R. This p.G488R substitution occurred in a highly conserved glycine residue of the mammalian DUOX2 protein. The two patients have different haplotypes, suggesting that the p.G488R alleles are the result of independent, recurrent mutations. Kasahara described a delayed-onset CH patient with two pathogenetic factors: a genetic defect and iodine excess [33]. Mutation screening for *DUOX2* identified biallelic mutations (p.E327X/p.H678R). This provided a new example of environmental modification of the CH phenotypes by genetic defects, which can potentially distort screening results.

In conclusion, we have identified two missense *DUOX2* mutations in Chinese patients with CH and goiter. In addition, we studied the functional significance of these mutants and revealed impaired H_2_O_2_ production and faster degradation in mutant DUOX2 proteins. Our findings confirm the genetic heterogeneity of *DUOX2* mutations. However, further studies are required to elucidate the underlying mechanisms.

## MATERIALS AND METHODS

### Patients

Ten unrelated CH patients (four boys, six girls, sex ratio 1:1.5, age 2.8±1.2 years) were recruited through the neonatal screening program in Shandong Province, China, between 2008 to 2013. Blood samples were collected from the heel and TSH levels were measured by enzyme-linked immunosorbent assay according to the same test standard. Subjects with elevated TSH (≥20 μIU/ml) levels were recalled for further evaluation. Serum TSH (normal range 0.27–4.2 uIU/ml) and free thyroxin (FT4; normal range 12–22 pmol/L) were determined by electrochemiluminescence assay. CH is diagnosed by high serum TSH levels and low FT4 levels. Ten children were diagnosed with CH and goiter through a 99mTc thyroid scan or thyroid ultrasound examination. They underwent diagnostic reevaluation at 2–4 years of age, based on thyroid hormone testing after 1 month of L-T_4_ withdrawal. Patients showing persistently high TSH values were defined as having permanent CH. To confirm the diagnosis of transient or permanent CH, all subjects were routinely examined. Blood samples were collected from patients only after obtaining written informed consent from their guardians and the study was approved by the Ethics Committee of the Affiliated Hospital of Qingdao University. All experiments were performed in accordance with the approved guidelines.

### DNA analysis

Genomic DNA was extracted from peripheral blood leukocytes using standard methods. *DUOX1*, *DUOXA1*, *DUOX2*, *TG* and *TPO* mutation screening was negative in all 10 subjects. The human *DUOX2* gene is encoded by 33 exons. Gene fragments covering the complete coding sequence, including intron-exon boundaries, of *DUOX2* (GenBank NM_014080.4) were amplified using the appropriate primer pairs for exons 1–33 of the *DUOX2*, as reported previously [24]. Identical amplification conditions were used in a total volume of 25 μl containing 250 nM dNTPs, 100 ng of template DNA, 0.5 μM of each primer, and 1.25 U AmpliTaq Gold DNA polymerase, in 1x reaction buffer (10 mM Tris HCl, pH 8.3, 50 mM KCl, 2.5 mM MgCl2). The samples were denatured at 94°C for 5 min followed by 35 cycles consisting of 30s at 94°C, 1 min at 51–65°C and 30s at 72°C, and a final primer extension of 10 min at 72°C. Amplified PCR products were purified and sequenced using the appropriate PCR primers and the BigDye Terminator Cycle Sequencing kit (Applied Biosystems, Foster City, CA, USA), and run on an automated sequencer, ABI 3730XL (Applied Biosystems), for mutational analysis.

### Construction of the expression vectors

cDNA was synthesized with reverse transcriptase M-MLV (Takara) by oligo(dT) priming of total RNA from a normal human thyroid gland. The *DUOX2* and *DUOXA2* open reading frames were amplified using native Pfu polymerase and cloned into pcDNA3.1 (Invitrogen). The primers for *DUOX2* were 5′-GGGGTACCCCATGCTCCGTGCAAGACCAGAG-3′ and 5′-GCTCTAGAGCTCAGAAGTTCTCATAGTGGTGC-3′ and the primers for *DUOXA2* were 5′-GGGGTACCCCATGACCCTGTGGAACGGCGTA-3′ and 5′-GCTCTAGAGCTCACAGGTTAGTGGTGATACA-3′. The two novel *DUOX2* mutations p.R354W and p.A1206T were introduced into the *DUOX2* expression vector by site-directed mutagenesis (Easy mutagenesis system, TransGen) using the following primers: p.R354W: F 5′-TGCCAGCTGTCATTTCTGGAAGGTCCT-3′ p.R354W:R 5′-AGAAATGACAGCTGGCATTTCTCATGTAG-3′ and p.A1206T: F5′-ATCATGTATGTCTTCACCTCCCACCA-3′ p.A120 6T:R5′-TGAAGACATACATGATGGCCAGGACC-3′. All constructs were confirmed by bidirectional DNA sequencing.

### Cell culture and transfection

Before transfection, cells were divided into eight groups according to the expression vector to be transfected, including *DUOX2* (containing *DUOX2* wild-type (WT) expression vector only), exon 10 (containing R354W *DUOX2* mutant expression vector only), exon 28 (containing A1206T *DUOX2* mutant expression vector only), *DUOX2* and *DUOXA2* (containing *DUOX2* WT expression vector and *DUOXA2* WT expression vector), exon 10 and *DUOXA2* (containing R354W *DUOX2* mutant expression vector and *DUOXA2* WT expression vector), exon 28 and *DUOXA2* (containing A1206T *DUOX2* mutant expression vector and *DUOXA2* WT expression vector), pcDNA3.1(containing an empty expression vector), and non transfected WT cells (no expression vectors). Hela cells and 293T cells were maintained in high-glucose Dulbecco's modified Eagle's medium supplemented with 100 U/ml penicillin, 100 ug/ml streptomycin, and 10% fetal bovine serum under humidified conditions with 5% CO_2_ at 37°C. The diluted DNA (200 ng *DUOX2* WT or *DUOX2* mutants with or without 40 ng *DUOXA2*) and the diluted transfection reagent (with 0.5μl Lipofectamine TM2000) were combined into DNA-Lipofectamine TM2000 complexes at room temperature for 20 minutes. When Hela cells and 293T cells reached 70−80% confluence, the DNA-Lipofectamine™ 2000 complexes were added into each well containing cells and medium and incubated at 37°C in a CO_2_ incubator for 6 hours, after which culture solution was replaced by complete culture medium.

### Measurement of H_2_O_2_ generation

Forty-eight hours after transfection, H_2_O_2_ production was determined in a reaction with cell-impermeable 10-acetyl-3,7-dihydroxyphenoxazine (Amplex Red reagent; Invitrogen) in the presence of excess peroxidase, producing fluorescent resorufin [34]. Briefly, cell monolayers were incubated in Dulbecco's phosphate-buffered saline (PBS) supplemented with 50 mM Amplex Red reagent and 0.2 U/ml H_2_O_2_ for 1 hour at 37°C. Relative fluorescence (excitation/emission, 535/595 nm) of the medium was measured and corrected for Amplex Red oxidation in wells containing nontransfected cells. A calibration curve was used to convert changes in fluorescence intensity into absolute nanomoles and the H_2_O_2_ concentration was maintained within the linear range of the standard calibration curve throughout. Renilla luciferase activity from co-transfected pRL-Tk plasmid (Promega) was used as internal control [23].

### Western blot analysis

Twenty-four hours after transfection, reaction termination was performed by 100 μg/mL cycloheximide (CHX; Sigma). Proteins were collected and extracted at different time intervals as follows: Cell pellets were washed once in PBS and resuspended in three volumes of CelLytic Lysis/Extraction Reagent (Sigma) with 1 mM PMSF. Cell suspension was incubated for 20 minutes on ice and cleared by centrifugation (12,000g, 15 minutes, 4°C). Total protein concentrations were determined by the Bradford method. Protein extracts were boiled in SDS/β-mercaptoethanol sample buffer. Samples (45 μg) were loaded into each lane of 8% polyacrylamide gels, separated by electrophoresis, and blotted onto polyvinylidene fluoride membranes (Amersham Pharmacia Biotech) by electrophoretic transfer. Western blotting was performed with a rabbit anti-DUOX2 antibody (Abgent) at 1:500 dilution, and bound antibody was detected using horseradish-peroxidase-conjugated goat anti-rabbit secondary IgGs. Detection by chemiluminescence reaction was performed using the ECL kit (Pierce, Appleton). Actin was detected as a loading control with a mouse anti-β-actin antibody (Santa Cruz). Three independent experiments were performed.

### Statistical analysis

All data are expressed as mean ± standard deviation (SD) and were analyzed by one-way analysis of variance followed by Newman–Keuls multiple comparison test as appropriate (GraphPad Prism version 5 software). A P value of <0.05 was considered statistically significant.
